# Enhancement of innate immunity in gingival epithelial cells by vitamin D and HDAC inhibitors

**DOI:** 10.3389/froh.2024.1378566

**Published:** 2024-03-14

**Authors:** Erika L. Figgins, Payal Arora, Denny Gao, Emily Porcelli, Rabab Ahmed, Carlo Amorin Daep, Garrett Keele, Lisa K. Ryan, Gill Diamond

**Affiliations:** ^1^ Department of Oral Biology, University of Florida College of Dentistry, Gainesville, FL, United States; ^2^ Department of Oral Immunology and Infectious Diseases, University of Louisville School of Dentistry, Louisville, KY, United States; ^3^ Global Technology Center, Colgate Palmolive Company, Piscataway, NJ, United States; ^4^ Division of Infectious Disease and Global Medicine, Department of Medicine, University of Florida College of Medicine, Gainesville, FL, United States; ^5^ Center for Predictive Medicine for Biodefense and Emerging Infectious Diseases, University of Louisville, Louisville, KY, United States

**Keywords:** anti-inflammatory, antiviral, antimicrobial peptide, vitamin D, periodontal

## Abstract

**Introduction:**

The human host defense peptide LL-37 is a component of the innate immune defense mechanisms of the oral cavity against colonization by microbes associated with periodontal disease. We have previously shown that the active form of vitamin D, 1,25(OH)_2_D_3_, can induce the expression of LL-37 in gingival epithelial cells (GEC), and prevent the invasion and growth of periopathogenic bacteria in these cells. Further, experimental vitamin D deficiency resulted in increased gingival inflammation and alveolar bone loss. Epidemiological studies have shown associations between vitamin D deficiency and periodontal disease in humans, suggesting application of vitamin D could be a useful therapeutic approach. Further, since we have shown the local activation of vitamin D by enzymes expressed in the GEC, we hypothesized that we could observe this enhancement with the stable, and inexpensive inactive form of vitamin D, which could be further increased with epigenetic regulators.

**Methods:**

We treated 3-dimensional primary cultures of GEC topically with the inactive form of vitamin D, in the presence and absence of selected histone deacetylase (HDAC) inhibitors. LL-37 mRNA levels were quantified by quantitative RT-PCR, and inhibition of invasion of bacteria was measured by fluorescence microscopy.

**Results:**

Vitamin D treatment led to an induction of LL-37 mRNA levels, as well as an inhibition of pro-inflammatory cytokine secretion. This effect was further enhanced by HDAC inhibitors, most strongly when the HDAC inhibitor, phenyl butyrate (PBA) was combined with Vitamin D_3_. This was observed both in solution and in a prototype gel formulation using sodium butyrate. Finally, this combination treatment led to an increase in the antimicrobial activity against infection by *Porphyromonas gingivalis* and *Filifactor alocis*, bacteria associated with periodontal lesions, as well as herpes simplex virus, which has also been shown to be associated with periodontal lesions.

**Conclusions:**

Our results demonstrate that a combination of inactive vitamin D and sodium butyrate could be developed as a safe treatment for periodontal disease.

## 1 Introduction

Periodontal disease is a chronic inflammatory disease of the gums and supporting tissues that if untreated, can lead to tooth loss and may also affect systemic health. It is associated with the colonization by specific keystone pathogenic bacteria, including *Porphyromonas gingivalis*, which leads to an inflammatory response and a dysbiosis of the commensal microbiota in the subgingival crevice [reviewed in (1)]. The initial innate immune defense against the microbial pathogens associated with periodontal disease includes a coordinated inflammatory response involving pro-inflammatory cytokines, and mediators, such as prostaglandin E_2_ (PGE2), as well as the regulated expression of a number of host defense peptides such as β-defensins and cathelicidins [reviewed in (2)]. The only human cathelicidin, LL-37, the product of the *CAMP* gene, is a multifunctional peptide, with antimicrobial activity against both Gram-positive and Gram-negative bacteria, as well as some viruses [reviewed in (3)]. In addition, it exhibits chemotactic properties and plays a role in dendritic cell maturation, identifying it as an important mediator in the innate and adaptive immune systems [reviewed in (4)]. Lack of LL-37 is associated with two human disorders, Morbus Kostman and Papillion-Lefevre Syndrome, in which there is severe periodontal disease associated with colonization by the periodontal pathogen *Aggregatibacter actinomycetemcomitans* (5–7). More recently, an association between aggressive periodontitis and mutations in the LL-37 gene has been identified (8). LL-37 gene expression can be induced by live bacteria (9), or by bacterial products such as LPS (10, 11), placing this peptide as part of the innate immune defense of the gingival epithelium.

LL-37 expression can also be induced by the hormonally active form of vitamin D, 1,25-dihydroxyvitamin D_3_ [1,25(OH)_2_D_3_] (12–15). This form is produced by two sequential hydroxylations of vitamin D. It has been generally accepted that this occurs initially by one of a number of 25-hydroxylases in the liver (leading to 25-hydroxyvitamin D_3_, or 25OHD_3_), and then by 25-hydroxyvitamin D1α-hydroxylase [1α-(OH)ase] in the kidney (16). However, recently, other cell types, including epithelial, breast, prostate and immune system cells (monocytes, macrophages and dendritic cells) were shown to produce the vitamin D activating 1α-hydroxylase (17, 18), suggesting that the active form of vitamin D can be produced locally. We have demonstrated that both steps can occur in oral epithelial cells (19). In addition to the induction of genes regulated through this pathway, vitamin D has also been shown to inhibit the induced expression of pro-inflammatory cytokines (20). This has been seen with LPS- and IL-1β-induced expression in gingival fibroblasts and periodontal ligament cells (21, 22), suggesting that vitamin D can play an important role in inhibiting the inflammation seen in periodontal disease. These data suggest that the application of inactive vitamin D to the oral epithelium can modulate the local immune system to improve gingival health.

Vitamin D deficiency is a factor associated with both the chronic and aggressive forms of periodontal disease in numerous populations (23–25). While the results of these studies vary, there is a large body of clinical data suggesting that vitamin D plays a role in the maintenance of periodontal health. Furthermore, recent animal experiments showed a reduction in alveolar bone loss by systemic supplementation of vitamin D in experimental models of periodontal disease in mice (26) and rats (27). We hypothesize that this relationship is due to the effect of vitamin D on both the innate immune activity of the gingival epithelium against periodontal pathogens to maintain microbial homeostasis.

We have previously demonstrated the regulation of the innate immune response in gingival epithelial cells (GEC) by 1,25(OH)_2_D_3_, including the induction of active LL-37 (28). Active vitamin D-mediated treatment of 3-dimensional cultures of the epithelial cells leads to an increase in antibacterial activity against *A. actinomycetemcomitans* on the mucosal surface of these cells, suggesting that vitamin D plays a role in the natural antibacterial defense against periodontal pathogens. We have also shown that treatment of both immortalized and primary cultures of GEC with 1,25(OH)_2_D_3_ leads to an increased defense against invasion by *P. gingivalis*, and an inhibition of expression of pro-inflammatory genes such as IL-6 and IL-1α (19). When mice were made deficient in serum vitamin D, they developed gingival inflammation and alveolar bone loss typical of periodontal disease (19). Together, these results support our hypothesis that vitamin D is crucial in the defense against the development of periodontal disease, at both the level of antibacterial defense and the inhibition of inflammation.

The use of the active form of vitamin D, while clearly demonstrating potential for enhancing periodontal host defense, is not therapeutically optimal (29). However, the LL-37 response to the stable and inexpensive inactive form of vitamin D is not very strong, and should we wish to develop a vitamin D-based therapy, we would like to enhance this response. Histone deacetylase (HDAC) inhibitors have been shown to enhance the transcriptional activity of vitamin D (30, 31), as well as the *CAMP* gene (32). One class of HDAC inhibitors, butyrate compounds, are known to activate the vitamin D receptor (VDR), by increasing the binding of PU.1 and cAMP response element binding protein (CREB1) to the *CAMP* promoter (33, 34), as well as inhibiting production of PGE2 (35). Thus, we hypothesize that these compounds could be useful in combination therapy with vitamin D as a method to induce LL-37 expression, as well as inhibiting the production of pro-inflammatory mediators. Here we examine the potential for direct delivery of vitamin D in its active and inactive forms, together with HDAC inhibitors, to enhance the innate defense against periodontal pathogens, and to enhance the antimicrobial response of oral epithelial cells.

## 2 Materials and methods

Cell cultures: The human oral keratinocyte cell line OKF6/TERT-1 was grown in keratinocyte serum free medium (KSFM) supplemented with L-glutamine and penicillin-streptomycin-fungizone (Sigma-Aldrich) in the presence of 0.03 M calcium chloride and bovine pituitary extract as described previously (19). Three-dimensional cultures of normal human gingival epithelial cells were obtained from MatTek. These represent confluent, terminally differentiated, multi-layer primary cultures of ginigival epithelium (EpiGingiva), obtained in 24-well transwell plates obtained from the producer (Supplementary Figure S1). After delivery, the transwells are transferred to 6-well plates into the medium provided, and the cultures are maintained in an air-liquid interface for 1–2 days prior to their use in the experiment.

### 2.1 HDAC inhibitors

All inhibitors were obtained from Sigma. Apicidin and valproic acid were dissolved in 100% ethanol at 10 mm and 100 mm respectively. Ellagic acid and MS-275 were dissolved in DMSO at 10 mm and 2.5 mm, respectively. Phenylbutyric acid and nicotinamide were dissolved in water at 1M and 0.2M, respectively. Inhibitors were added to cultures to the following final concentration: apicidin, 10 µm; valproic acid, 100 µm; ellagic acid, 10 µm; MS-275, 2.5 µm; PBA, 1 mm; nicotinamide, 200 µm.

### 2.2 Vitamin D

Vitamin D_3_, 25(OH)D_3_ and 1,25(OH)_2_D_3_ (Sigma) were dissolved in 100% ethanol at 10^−5^M and kept in the dark at −20°C. Stocks were freshly diluted to 10^−7^ M in either sterile medium or phosphate buffered saline prior to each use. Control vehicle was 0.1% ethanol in sterile medium or PBS. We observed no toxic effect of either the vitamin D metabolites at this concentration, nor of the vehicle control as measured by MTT assay (data not shown).

### 2.3 ELISA

PGE2 levels were quantified by ELISA (InVitrogen) using conditions provided by the supplier.

### 2.4 Bacterial invasion assays

*Filifactor alocis* ATCC 35896 was cultured in brain heart infusion as previously described in (36). *Porphyromonas gingivalis* ATCC 33277 was cultured in Tryptic soy broth, supplemented with 5 μg/ml hemin, 250 μg/ml L-cysteine and 1 μg/ml menadione as described previously (37). Bacteria were stained with carboxyfluorescein diacetate succinimidyl ester (CFSE) at a final concentration of 100 µm for 15 min at 37°C, followed by two washes in PBS. Bacteria were resuspended in antibiotic-free basal medium. *F. alocis* were added to cultured OKF6/TERT-1 cells in chamber slides at an MOI of (200:1) for 90 min, followed by washing the cultures to remove extracellular bacteria. Cultures were stained with DAPI and Texas Red to identify the cells, and were imaged by confocal microscopy. Three to five images in each of triplicate cultures were assessed for efficiency of invasion, which was calculated by dividing the number of cells positive for the presence of bacteria by the total number of cells in the image.

### 2.5 Quantification of mRNA

Total nucleic acids were extracted from tissue culture GECs with QiaShredder spin columns and the RNAeasy Plus kit (Qiagen) according to the manufacturer's guidelines. BioRad's iScript cDNA library kit was used according to the supplied directions. Relative mRNA levels were measured with a SsoAdvanced Universal SYBR Green Supermix (BioRad) on a BioRad CFX96 Touch Real-Time PCR Detection System thermal cycler in 96-well plates and quantified according to the 2^−ΔΔCT^ method, relative to β-actin as a housekeeping gene, and control-treated cultures or tissue. Primer sequences are: *CYP24A1* (F: 5′-AGTATCTGCCTCGTGTTGTATG-3′; R: 5′-GTGGCCTGGATGTCGTATTT-3′); *CAMP* (F:5′-CCCTGCTGGGTGATTTCTT-3′; R:5′ GTGGCCTGGATGTCGTATTT-3′); β-actin (F:5′-GGATCAGCAAGCAGGAGTATG-3′; R:5′ AGAAAGGGTACGCAACTAA-3′).

### 2.6 Quantification of HSV-1 DNA

HSV-1 (GFP strain) was propagated in VERO cells as described (38). OKF-6/TERT-1 cells were infected at an MOI of 0.1:1 for 24 h. After infection, cells were harvested and homogenized using Qiagen QIAshredder protocol. The obtained lysate was then used to extract RNA and DNA using the Qiagen RNeasy Plus Micro kit. Once RNA was extracted using the manufacturer protocol, 50 µl of 8.0 mm NaOH was added directly to membrane. Tubes were then incubated for 10 min at 55°C. DNA was eluted by centrifugation for 3 min at 5,000 × g. qPCR was performed on a BIO RAD myCycler using SsoAdvanced Universal SYBR green supermix (Bio-RAD #1725274), using 1.5 µl cellular DNA per reaction, with HSV UL30 primers (F: 3′-AGAGGGACATCCAGGACTTTGT-5′; R: 3′-CAGGCGCTTGTTGGTGTAC5′). DNA levels were normalized to β-actin in each sample and the levels determined using the 2^−ΔΔCT^ method compared to ethanol-treated cells.

### 2.7 Statistical anlysis

Experiments were carried out in triplicate, and repeated at least once for reproducibility. Data were analyzed using GraphPad Prism 10 with unpaired *t*-tests, one and two-way analysis of variance (ANOVA) with post-hoc testing using Tukey's multiple comparisons test.

## 3 Results

### 3.1 Effect of HDAC inhibitors on vitamin D-mediated induction of gene expression

**Figure 1:**
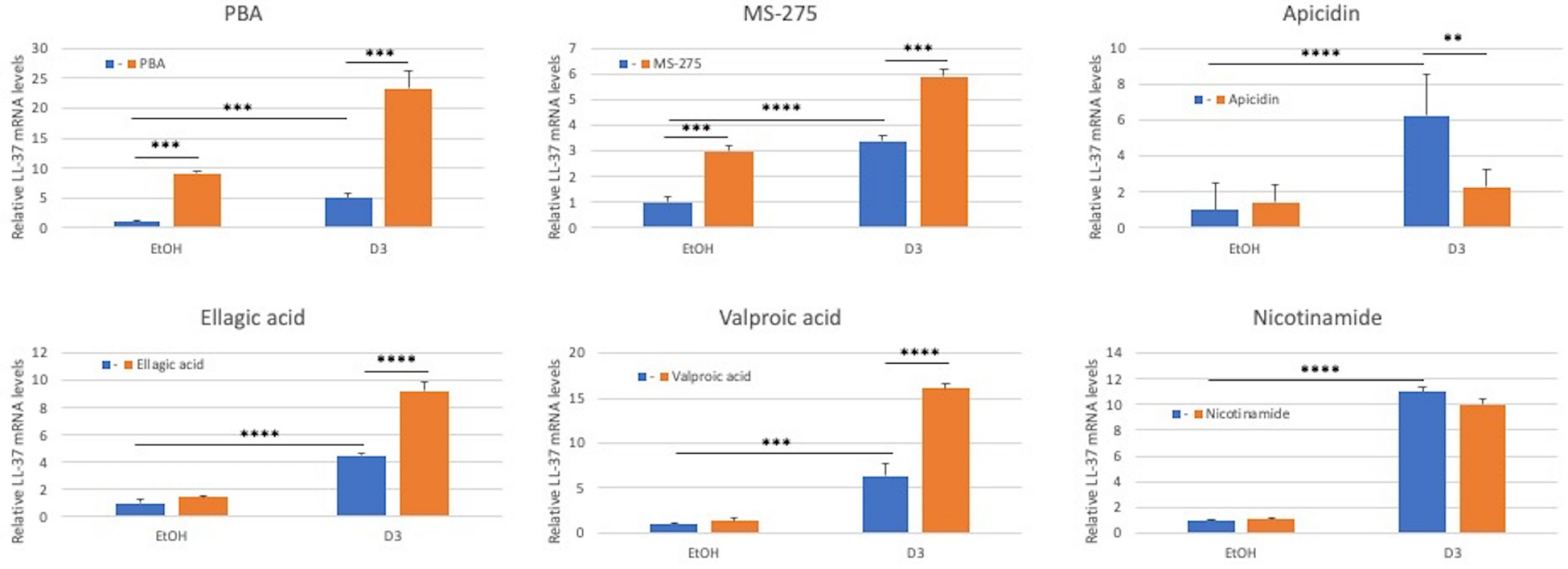
Effect of HDAC inhibitors on inactive vitamin D_3_-mediated induction of LL-37 gene expression in oral keratinocytes. OKF6/TERT-1 cells were incubated with either 0.1% Ethanol (EtOH) in the presence or absence of 1 µm inactive vitamin D3 for 24 h. Added to each was HDAC inhibitors at the following concentrations: PBA (1 mm); MS-275 (2 mm); Apidacin (1 µm); Ellagic acid (2 mm); Valproic acid (100 µm); and Nicotinamide (2 mm). Total mRNA was isolated and relative LL-37 mRNA levels were quantified by QRT-PCR relative to β-actin. Data are shown as mean (*n* = 3) ±SD. *p < 0.05; **p < 0.005; ***p < 0.0005; ****p < 0.00005 by 2-way ANOVA and Tukey's post-hoc test.

Since inactive vitamin D treatment can regulate expression of LL-37 (19), albeit at a lesser efficiency than does active 1,25(OH)_2_D_3_ (19), we wished to examine the potential to enhance this response. We treated OKF6/TERT-1 cells with vitamin D_3_ or in combination with HDAC inhibitors for 24 h, followed by quantification of LL-37 mRNA. Specifically, we used nicotinamide (an inhibitor of Class III HADACs); phenylbutyrate (PBA) and valproic acid (inhibitors of Class I and Class IIa HDACs); apicidin (a class 1 HDAC inhibitor), and MS-275 (an inhibitor of HDACs 1-III). We also tested ellagic acid, which is an inhibitor of coactivator associated arginine methyltransferase 1 (CARM1). The results shown in Figure 1 demonstrate that inactive vitamin D_3_ at 1 µm was sufficient to induce LL-37 mRNA levels from 5 to 10 fold, as did both MS-275 and PBA alone. Further, MS-275, PBA, valproic acid and ellagic acid all enhanced the expression of LL-37 when combined with vitamin D_3_. Nicotinamide had no effect, either alone or in combination with vitamin D_3_.

### 3.2 Effect of butyrate on vitamin D_3_-mediated gene expression

**Figure 2:**
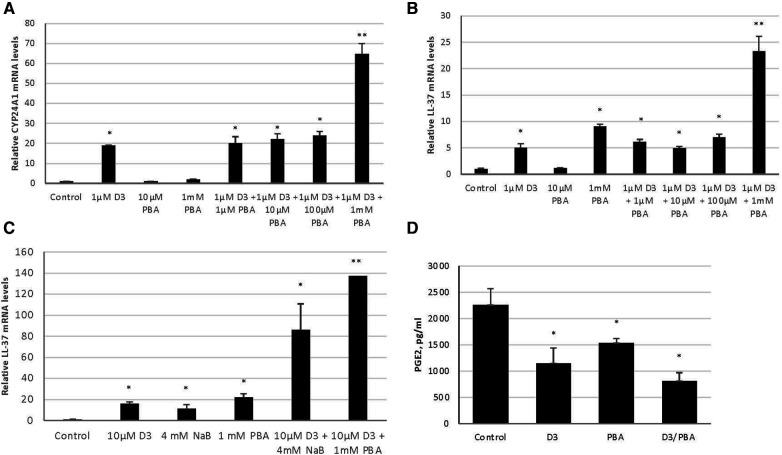
Synergistic effect of vitamin D_3_ and butyrate compounds on LL-37 and PGE2 in 3D cultures of gingival epithelial cells. OKF6/TERT-1 cells (**A,B**) or 3D air-liquid interface EpiGingiva cultures (**C,D**) were treated with vitamin D_3_, PBA or NaB, or a combination for 24 h. Vitamin D_3_ and butyrate were added to the medium in (**A,B**), and in 10 µl on the apical surface for (**C,D**). Total mRNA was isolated and relative LL-37 mRNA levels were quantified by QRT-PCR. Basolateral medium was removed, and PGE2 concentrations were quantified by ELISA (**D**) Data are shown as mean (*n* = 3) ±SD. *p < 0.05; **p < 0.005, by *t*-test.

Considering all of the HDAC inhibitors tested, PBA provided the greatest enhancement of vitamin D_3_-mediated LL-37 expression. To examine this effect further, we carried out a dose-response study of PBA between 100 µm and 1 mm. The results in Figure 2A show a dose-dependent enhancement of vitamin D_3_ induction of the VDR-mediated gene *CYP24A1*. We see a similar response of LL-37 (Figure 2B), indicating that this combination could be used to induce LL-37 in tissues. One main difference, however, is in response to PBA alone. There is no induction of CYP24A1 to PBA, while 1 mm PBA treatment led to an induction of LL-37.

To examine the effect of topical delivery, we repeated the experiments using administration of vitamin D and butyrate to the apical surface of 3D cultures (Supplementary Figure S1). The results in Figure 2C show an induction of LL-37 expression in response to topical administration of both 10 µm Vitamin D_3_ and 1 mm PBA. However, since PBA is not well-tolerated for topical oral applications, we repeated the experiment using sodium butyrate (NaB). The results show a similar LL-37 induction, with NaB enhancing the vitamin D_3_ response in topical application to 3D cultures both alone, and in synergy with vitamin D (Figure 2C). Furthermore, we also see a concomitant reduction of the pro-inflammatory mediator PGE2, as measured by ELISA of the basolateral medium (Figure 2D).

### 3.3 Delivery of vitamin D and PBA in a complex system

**Figure 3:**
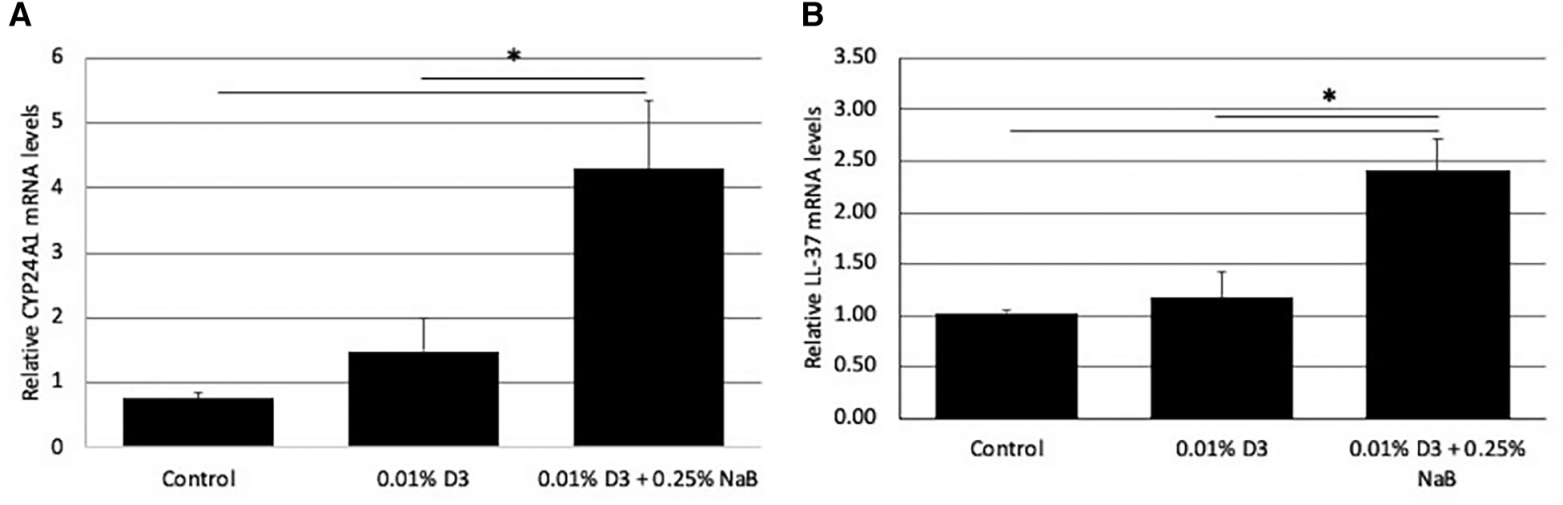
Effect of gel formulations with vitamin D_3_ on gene expression. Gels containing vitamin D_3_, or a combination of vitamin D and NaB at the concentrations shown were added to OKF6/TERT1 cells for 10 min, rinsed, then the cultures were incubated in fresh medium for a further 6 h. Relative mRNA was quantified for CYP24A1 (**A**) and LL-37 (**B**) levels by QRT-PCR. Data are shown as mean (*n* = 3) ±SD. *p < 0.05 by *t*-test.

Since a topical treatment *in vivo* would require a much shorter challenge with the inducing agent than the *in vitro* studies above, we developed gel formulations containing a high concentration of vitamin D_3_ alone (0.01%w/v, which equals 260 µm), or in combination with NaB (0.25%w/v, 227 mm). To quantify the effect of a short-term treatment with these formulations, we treated OKF6/TERT cells with these gels for 10 min. After the treatment, the cultures were washed twice with fresh medium, and incubated for a further 3 h in medium without vitamin D_3_. Total mRNA was isolated, and the response of *CYP24A1* and LL-37 were quantified by qRT-PCR. The results in Figure 3 show that even a very short treatment with a supra-physiological concentration of vitamin D together with NaB was sufficient to lead to a rapid response of the cells. The combination of vitamin D and NaB induced a significant increase in LL-37 expression when compared to either control or vitamin D treatment alone.

### 3.4 Effect of butyrate and vitamin D on microbial infection

**Figure 4:**
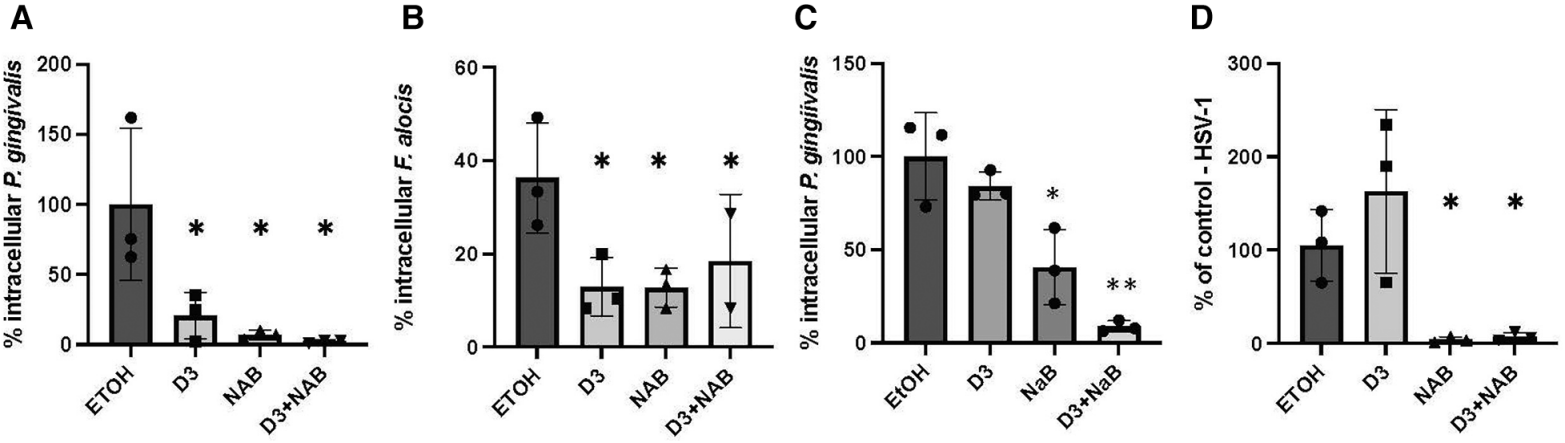
Inhibition of microbial infection by pretreatment with vitamin D and butyrate. (**A,B**) Bacterial infection. OKF6/TERT-1 cells were incubated for 24 h with 100 nm Vitamin D_3_, 2 mm Sodium butyrate (NaB), or a combination of 2 mm sodium butyrate and 100 nm Vitamin D_3_. After 24 h, cells were infected with either *P. gingivalis* (**A**) or *F. alocis* (**B**) at an MOI of 500:1 or 200:1, respectively. Bacteria were detected by fluorescent microscopy using 400 µg/ml CFSE and % infected was calculated. (**C**) Intracellular vs. extracellular killing. OKF6/TERT-1 cells were incubated for 24 h with 100 nm 1,25(OH)_2_D_3_, 2 mm NaB, or a combination of butyrate and 1,25(OH)_2_D_3_. Medium was removed, cells were washed once, and infected with *P. gingivalis* at an MOI of 500:1. Intracellular bacteria were quantified after 2 h. (**D**) Viral infection. OKF6/TERT-1 cells were incubated for 24 h with 100 nm Vitamin D_3_, 2 mm Sodium butyrate (NaB), or a combination of 2 mm sodium butyrate and Vitamin D_3_. After 24 h, cells were infected with HSV-1 at an MOI of 0.1:1. After 24 h, total DNA was isolated and relative HSV-1 DNA levels were quantified by QPCR normalized to genomic β-actin DNA. Data are shown as mean (*n* = 3) ±SD. *p < 0.05; **p < 0.005, by *t*-test.

To demonstrate a protective effect of vitamin D and butyrate treatment on oral epithelial cells against microbial challenge, we pre-treated OKF6/TERT cells with Vitamin D_3_ in the presence or absence of 2 mm sodium butyrate, followed by infection with microbial challenge. Both *P. gingivalis* and *F. alocis* are associated with the development of periodontitis, and have been shown to invade gingival epithelial cells (39). The results in Figures 4A,B show that both vitamin D_3_ alone and NaB alone were sufficient to reduce the invasion of bacteria. There was a further reduction of *P. gingivalis* by the combination, to 2.3% of control, which was not observed with *F. alocis*.

To determine whether the increase in killing was due to secretion of antibacterial factors, such as LL-37, or to an increase in intracellular killing, we repeated the experiment, but removed the medium after the vitamin D/NaB treatment prior to bacterial challenge. The results in Figure 4C show that removal of the vitamin D-treated medium resulted in an inhibition of the reduction in intracellular *P. gingivalis*, suggesting that a significant portion of the vitamin D treatment leads to the prevention of invasion by extracellular killing. However, removal of the medium after treatment with NaB led to a 60% inhibiton of invasion by *P. gingivalis*.

Herpesviruses are often associated with periodontal lesions, so we quantified the combined effect of vitamin D and butyrate treatment on infection of oral epithelial cells with HSV-1. The results in Figure 4D show no reduction of HSV-1 infection by Vitamin D_3_ alone, however NaB was sufficient to almost completely prevent HSV-1 infection. The combination of Vitamin D_3_ and NaB gave a similar inhibitory response to NaB alone.

## 4 Discussion

Vitamin D deficiency is associated with periodontal disease in human populations, as determined by a meta-analysis of numerous worldwide studies (40). We also demonstrated an association between vitamin D deficiency and characteristics of periodontal disease in experimental mice, including increased inflammation in the gingiva, and alveolar bone loss (19). Numerous studies have shown that the active form of vitamin D, 1,25(OH)_2_D_3_, can induce the expression of LL-37, and inhibit the expression of pro-inflammatory cytokines in gingival epithelium [reviewed in (41)]. The intermediate inactive form, 25OHD_3_, has also been shown to carry out this activity, as the activating enzyme, 1-α hydroxylase (encoded by the *CYP24A1* gene) is expressed in oral epithelial cells (28), gingival fibroblasts and periodontal ligament cells (42, 43). The vast majority of research into the activity and effects of vitamin D in human health has focused on systemic use, usually by oral supplementation, as vitamin D is very safe, even at high doses, and is easily absorbed and stored in the body (44). This has led to mixed results, most likely because oral supplementation can only increase serum 25OHD_3_ levels [and tissue levels of 1,25(OH)_2_D_3_] to a limited degree. Similarly, Gui et al. (45) showed that while systemic administration of 1,25(OH)_2_D_3_ (by daily intraperitoneal injection over two weeks) initially led to reduced inflammation, there was increased inflammation in the long term. Since others have shown that HDAC inhibitors can enhance the effect of 1,25(OH)_2_D_3_ (46–48), and we have demonstrated that the gingival epithelial cells are capable of converting the inactive cholecalciferol form to the active form, we hypothesized that a combination of inactive vitamin D_3_ and HDAC inhibitors could bolster the LL-37-specific innate immune defense against oral pathogens that can lead to gingival inflammation and other infectious conditions.

Since oral supplementation together with HDAC inhibitors is not a viable option for periodontal disease, we tested a topical administration *in vitro*, using 3D-cultures of gingival epithelial cells. Our results indicate that the combination of a supraphysiological concentration of inactive vitamin D_3_ and butyrate compounds was sufficient to enhance LL-37 gene expression in these cultures. LL-37 expression was also enhanced with the combinations of the active form of vitamin D and buyrate (data not shown). While topical administration of active vitamin D_3_ analogues has been used in combination with butyrate compounds to treat dermatological conditions [see, for example, (49)], this is the first demonstration that it could be used for topical oral applications to improve periodontal disease.

The induction of LL-37 gene expression in cultured GECs by vitamin D_3_ occurs at a lower level of efficiency than 25OHD_3_ or 1,25(OH)_2_D_3_ (19). This is most likely due to the inefficiency of the epithelial cell-mediated two-step activation of vitamin D. While the physiological concentration of active vitamin D_3_ is approximately 10 nm, for most experiments we increased the concentration of vitamin D3 to 10 µm, to account for low efficiency, which provided a similar level of gene induction. However, since it is known that HDAC inhibitors can enhance the effect of VDR-mediated LL-37 gene expression (31, 32, 50), we tested several inhibitors for their ability to enhance the effect of inactive vitamin D_3_ in the induction of LL-37 in gingival epithelial cell cultures. Our results clearly demonstrate that while inactive vitamin D_3_ can induce the expression of LL-37, this is regulated by several classes of HDACs, as is evidenced by the synergistic enhancement of four of the six inhibitors tested, which affect different classes of HDACs. As these inhibitors affect different classes of HDACs, this suggests that the vitamin D-mediated induction of LL-37 is complex, involving several mediators of transcription. Furthermore, the enhancement by ellagic acid suggests that the regulation also involves histone methylation via CARM1, similar to the regulation of CYP24A1 by 1,25(OH)_2_D_3_ (51). Further, addition of butyrate compounds was able to enhance this activity in both submerged cultures and with topical application to the apical surface of 3D-cultures to a great extent. Phenylbutyric acid (PBA) is an epigenetic modifier that enhances the LL-37 transcriptional activity of vitamin D_3_, although its activity may be through multiple mechanisms (52, 53), and thus its use as a therapeutic agent to act in synergy with vitamin D_3_ currently being investigated (54). Interestingly, one inhibitor, apicidin, reduced the effect of vitamin D on LL-37 expression, suggesting that its regulation of vitamin D-mediated gene expression can occur in both directions.

Periodontal disease is characterized by a dysbiosis of the gingival crevice, with *P. gingivalis* often considered a primary player in microbiota of the periodontal lesion. Our previous data has shown that pre-treatment of primary cultures of gingival epithelial cells with 1,25(OH)_2_D_3_ leads to a reduction in intracellular *P. gingivalis*, suggesting that the induced LL-37 enhances the innate antibacterial defense of the cell (19). Other pathogens are also found associated with the periodontal lesion, including *F. alocis* (55). Our data here show that we can recapitulate this response against *P. gingivalis* and *F. alocis* with both inactive vitamin D and butyrate. In addition, we observe a difference in their activities, in that removal of the extracellular medium after stimulation prevents this killing, suggesting that the activity is due to secretion of LL-37. However, there is still a significant amount of NaB-stimulated killing that occurs intracellularly. This suggests that butyrate may stimulate the production of other antimicrobial agents that may work intracellularly.

Members of the herpesvirus family are also found associated with periodontal lesions [reviewed in (56)]. To demonstrate that induction of LL-37 leads to a broad-spectrum antimicrobial enhancement of activity, we also treated oral epithelial cells with vitamin D_3_ and NaB to show a reduction in infection by both *F. alocis* and HSV-1 in the presence of NaB. The reduction of viral infection was similar to the pre-incubation of purified virus with 20 µg/ml LL-37 prior to infection in oral epithelial cells (57), a result similar those observed with LL-37 against HSV-1 infection of ocular epithelial cells (58). Similarly, we demonstrated that LL-37 could inactivate a related virus, Kaposi's Sarcoma-associated Herpes Virus (KSHV) through disruption of the viral envelope (59). We speculate that the induction of LL-37 is inactivating HSV-1 through the same mechanism, and would work together with the other innate immune antiviral defense mechanisms of the oral cavity (60). A similar observation has been made that LL-37 can transport the microbial DNA sensor cGAMP to enhance antiviral innate immunity (61). Others have demonstrated that treatment of cultured airway epithelial cells with 1,25(OH)_2_D_3_ can lead to an inhibition of infection by respiratory syncytial virus, and influenza virus infection, and the associated inflammatory responses (45, 62–64). However, since our observations indicate that NaB is sufficient for induction of antiviral activity, a non-LL-37 based mechanism is also possible. The butyrate-secreting probiotic bacterium *Clostridium butyricum* has been shown to induce antiviral activity through the induction of antiviral and interferon-related genes (65). This suggests that, like our observations with bacteria, butyrate can induce multiple antimicrobial pathways. Our current results suggest the potential use of inactive topical vitamin D_3_ in combination with an HDAC inhibitor, such as NaB, in as an adjunctive therapy for periodontal disease.

## Data Availability

The raw data supporting the conclusions of this article will be made available by the authors, without undue reservation.

## Supplementary material

Supplementary Figure S1 Three-dimensional model of gingival epithelium. EpiGingival cultures obtained from Mattek, Inc. are grown on porous filter inserts, maintained in basolateral serum-free medium. Vitamin D treatments are applied to the apical surface.
