# Supplementary figures and images for: Enhancement of innate immunity in gingival epithelial cells by vitamin D and HDAC inhibitors
Source: Front Oral Health. 2024 Mar 14;5:1378566. doi: 10.3389/froh.2024.1378566 (PMC10986367; doi:10.3389/froh.2024.1378566)

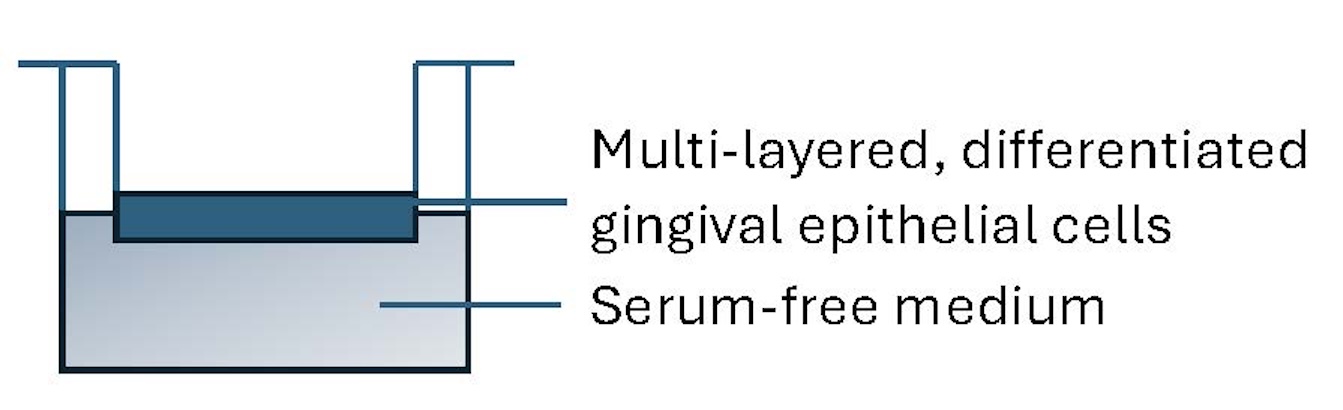

Supplement: Supplementary Figure S1 — Three-dimensional model of gingival epithelium. EpiGingival cultures obtained from Mattek, Inc. are grown on porous filter inserts, maintained in basolateral serum-free medium. Vitamin D treatments are applied to the apical surface. [file Image1.jpeg]
